# Elucidating the Color of Rosé Wines Using Polyphenol-Targeted Metabolomics

**DOI:** 10.3390/molecules27041359

**Published:** 2022-02-17

**Authors:** Cécile Leborgne, Marine Lambert, Marie-Agnès Ducasse, Emmanuelle Meudec, Arnaud Verbaere, Nicolas Sommerer, Jean-Claude Boulet, Gilles Masson, Jean-Roch Mouret, Véronique Cheynier

**Affiliations:** 1 SPO, INRAE, Univ. Montpellier, Institut Agro, 34060 Montpellier, France; cecile.leborgne@vignevin.com (C.L.); marine.lambert@gmail.com (M.L.); emmanuelle.meudec@inrae.fr (E.M.); arnaud.verbaere@inrae.fr (A.V.); nicolas.sommerer@inrae.fr (N.S.); jean-claude.boulet@inrae.fr (J.-C.B.); jean-roch.mouret@inrae.fr (J.-R.M.); 2 Institut Français de la Vigne et du Vin, Centre du Rosé, 83550 Vidauban, France; gilles.masson@vignevin.com; 3 INRAE, PROBE Research Infrastructure, Polyphenol Analytical Facility, 34060 Montpellier, France; 4 Institut Français de la Vigne et du Vin, UMT OENOTYPAGE, Domaine de Pech Rouge, 11430 Gruissan, France; marie-agnes.ducasse@vignevin.com

**Keywords:** wine, rosé wine, color, polyphenols, anthocyanins, metabolomics, UHPLC, mass spectrometry, Multiple Reaction Monitoring—MRM, chemometrics

## Abstract

The color of rosé wines is extremely diverse and a key element in their marketing. It is due to the presence of anthocyanins and of additional pigments derived from them and from other wine constituents. To explore the pigment composition and determine its links with color, 268 commercial rosé wines were analysed. The concentration of 125 polyphenolic compounds was determined by a targeted metabolomics approach using ultra high-performance liquid chromatography coupled to triple quadrupole mass spectrometry (UHPLC-QqQ-MS) analysis in the Multiple Reaction Monitoring (MRM) mode and the color characterised by spectrophotometry and CieLab parameters. Chemometrics analysis of the composition and color data showed that although color intensity is primarily determined by polyphenol extraction (especially anthocyanins and flavanols) from the grapes, different color styles correspond to different pigment compositions. The salmon shade of light rosé wines is mostly due to pyranoanthocyanin pigments, resulting from reactions of anthocyanins with phenolic acids and pyruvic acid, a yeast metabolite. Redness of intermediate color wines is related to anthocyanins and carboxypoyranoanthocyanins and that of dark rosé wines to products of anthocyanin reactions with flavanols while yellowness of these wines is associated to oxidation.

## 1. Introduction

Rosé wine consumption has increased greatly in the last 20 years, reaching over 10% of the global market of still wines [1]. Color is an essential element in the perception of rosé wine quality. As these wines are usually sold in clear glass bottles, it is also a major driver of consumer purchases, although there is no consensus on the ideal color [1]. Rosé wine color varies widely from very pale “sand” or “nacre” to vivid “garnet” nuances, through orange “apricot” or red “raspberry” shades, depending on the grape cultivar and on various factors linked to environment, vine growing practices and wine-making process.

The color of rosé wine, like that of red wines, is due to the presence of anthocyanins extracted from red grape skins and of additional pigments formed from them or through reactions of other must and wine components during wine making and ageing. Genuine anthocyanins are usually described as red flavylium cations but this form is predominant only at very low pH values and anthocyanins are present mostly in their colorless hydrated hemiketal form [2,3] under the pH conditions encountered in wine. Moreover, flavylium cations react with sulfite to form colorless bisulfite adducts [4], a reaction referred to as sulfite bleaching [5]. Both of these reactions are reversible, so the red flavylium cation can be released from colorless water or sulfite adducts. In particular, the hydration equilibrium can be shifted to the red flavylium forms in the presence of copigments such as flavonols, flavanols and hydroxycinnamic acids [3]. This phenomenon is responsible for 30–50% of the color in young red wines, but may be limited in rosé wines by the low pigment concentration. Other reactions of anthocyanins described in red wines include formation of red flavanol-anthocyanin adducts [6,7] and colorless anthocyanin–flavanol adducts [8], condensation with acetaldehyde yielding purple methylmethine linked (ethyl-linked) adducts, and reactions with hydroxycinnamic acids or vinylphenols and with carbonyl compounds such as acetaldehyde and pyruvic acid, yielding different groups of orange pyranoanthocyanins [9,10,11].

Limited data is available on the composition of rosé wines. A study performed on Grenache rosé wines [11] provided quantitative analysis of 28 polyphenols, showing that their proportions were very different from those encountered in red wines made from the same grape variety [12]. In these rosé wines, the major phenolic compounds were hydroxycinnamic acids, while anthocyanins and flavanols were present in lower amounts. Higher color intensity and pinker shade were related to higher concentrations of anthocyanins and phenolic acids and lower pH values [11]. Particular pigment profiles were pointed out, with a lower proportion of coumaroylated derivatives, likely reflecting poor extraction rates of these hydrophobic pigments compared to other anthocyanins, and higher proportions of derived pigments, including several groups of pyranoanthocyanins. This study also provided the first report of anthocyanin–caftaric acid adducts in wines. Detection of these pigments, resulting from nucleophilic addition of anthocyanins onto caftaric acid quinones formed by enzymatic oxidation of caftaric acid [13], may reflect the fact that rosé wine-making technology enables enzymatic oxidation at pressing and/or the high levels of hydroxycinnamic acids in the Grenache variety. More recently, a method based on ULPC-MS/MS analysis in the multiple reaction monitoring mode was developed for quantitative determination of 152 phenolic compounds in rosé wines [14]. Application of this method to twelve rosé wines showed differences in phenolic composition, and especially in the concentrations of hydroxycinnamic acids and of anthocyanins, flavanols, and gallic acid, suggesting an impact of both varietal characteristics and wine-making process (enzymatic oxidation, extraction).

Changes in color [15] and/or phenolic composition [11,16] of rosé wines induced by wine processing and storage have also been described but the relationships between phenolic composition and color are only partly understood. The objective of the present work was to explore the phenolic composition of rosé wines and explain differences in color styles. This was achieved on a large collection of commercial wines reflecting the diversity of rosé wine styles worldwide, using semi-quantitative phenolic composition data obtained by targeted HPLC-MS/MS in the MRM mode, along with classical oenological data (e.g., pH, sulfite concentration), and color intensity, hue values, and L*a*b* CIELAB data determined by spectrophotometry.

## 2. Results

### 2.1. Rosé Wine Diversity

Two hundred and sixty-eight commercial rosé wines were selected from the Rosé du Monde collection 2011, so as to obtain a diverse set of color styles and origins. These wines were analyzed by targeted UHPLC-QqQ-MS in the MRM mode targeting phenolic composition. Enological and color data were also collected.

The variable list and codes used and the data presented in this study are provided as Supplementary Materials (Table S1 and Table S2, respectively).

#### 2.1.1. Enological Parameters and Color

Enological data showed large diversity within the Rosé du Monde Collection, with pH values ranging from 2.92 to 3.98, percent alcohol from 9.75 to 18.77, total sulfite content from 18 mg·L^−1^ to 237 mg·L^−1^ and free sulfite content from 5 mg·L^−1^ to 132 mg·L^−1^.

**Figure 1:**
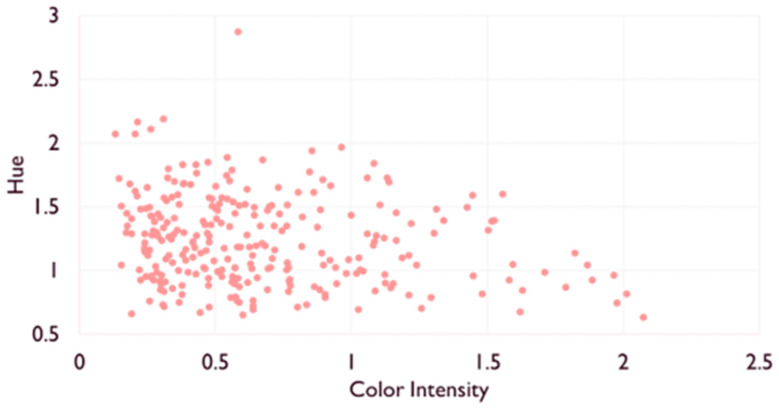
Scatterplot of color intensity and hue values of the 268 wines.

Wine color analysis also revealed large diversity, with a broad range of color intensities from light (CImin = 0.15) to dark (CImax = 2.07) and hues from reddish (Hmin = 0.63) to orange (Hmax = 2.87) due to different wine-making techniques or grape varieties (Figure 1). Even if Hue and CI were not correlated, the highest values of Hue were observed for the lowest CI. CI was strongly correlated with both A420 (R^2^ = 0.98) and A520 (R^2^ = 0.99) which were also correlated together (R^2^ = 0.94). Accordingly, L* values ranged from 57.6 to 97.3, a* values from −0.88 to 50.9, and b* values from 5.02 to 31.59. L* showed strong negative correlation with CI (R^2^ = −0.99) and a* (R^2^ = −0.98), which were positively correlated together (R^2^ = 0.96). The yellow component b* was also negatively correlated with L* (R^2^ = −0.75) and positively correlated with a* (0.64) and CI (R^2^ = 0.78), the latter correlation being mostly driven by A420 (R^2^ = 0.88).

#### 2.1.2. Phenolic Composition

Targeted MS analysis showed large composition differences between the rosé wine samples. The most abundant classes of phenolic compounds in rosé wines were flavanols, hydroxycinnamic acids, benzoic acids, and anthocyanins, aromatic amino acids (tyrosine and tryptophan), alcohols derived from these amino acids (i.e., tyrosol, hydroxytyrosol, and tryptophol), while stilbenes, flavonols, and dihydroflavonols were present in lower amounts but large variations were observed in all families.

Major hydroxycinnamic acid derivatives in the rosé wines were hydroxycinnamoyl tartaric acids, namely caftaric acid (0.8–83.1 mg·L^−1^), coutaric acid (1.1–36.9 mg·L^−1^), and GRP (0.1–82. 7 mg·L^−1^). Rather large amounts of free hydroxycinnamic acids (up to 28.9 mg·L^−1^ and 6.8 mg·L^−1^, for caffeic acid and *p*-coumaric acid, respectively) were detected in some wines, suggesting some hydrolysis.

Flavanols were represented mainly by monomers, i.e., catechin (0.3–36 mg·L^−1^) and epicatechin (0.1–100.6 mg·L^−1^), along with lower amounts of dimers, and especially B1 (up to 48.7 mg·L^−1^) and B2 (up to 11.7 mg·L^−1^), and small amounts of trimers. Concentrations of flavanol units measured after phloroglucinolysis ranged between 1 and 258.8 mg·L^−1^. Their total concentration exceeded the sum of monomers and oligomers in most samples, indicating the presence of larger oligomers and /or tannin derivatives. Mean degree of polymerisation (mDP) values ranging from 1.2 to 7 were calculated from the phloroglucinolysis data.

Major anthocyanins were malvidin-3-O-glucoside (0.04–42.9 mg·L^−1^) and malvidin-3-O-acetylglucoside (0–20.1 mg·L^−1^), followed by peonidin 3-O-glucoside (0.04–11.02 mg·L^−1^), and malvidin-3-O-coumaroylglucoside (0–8. 9 mg·L^−1^). Gallic acid was the most abundant benzoic acid with values ranging from 0.5 to 41.3 mg·L^−1^. Stilbenes were mostly represented by *cis*- and *trans*-piceid (maximum values at 21.8 and 12.6 mg·L^−1^, respectively). Major flavonols (0–15 mg·L^−1^) were quercetin derivatives along with lower amounts of myricetin glycosides while astilbin and taxifolin were the only dihydroflavonols detected.

Anthocyanin derivatives were detected in lower amounts but also showed large variations. The most abundant derived pigments were those formed by reactions involving acetaldehyde, i.e., pyranoanthocyanins (Py) flavanol-methylmethine-anthocyanins (flavanol-ethyl-anthocyanins, FEA), and flavanyl-pyranoanthocyanins (FPy), present in lower amounts. Other important families included carboxypyranoanthocyanins (CP, phenylpyranoanthocyanins (catechyl-, hydroxyphenyl-, guaiacyl-, and syringyl-pyranoanthocyanins, PP) and flavanol-anthocyanin adducts (F-A). Trace amounts of caftaric-anthocyanin adducts (CAF) and of A-type anthocyanin-flavanol adducts (A-F) were also detected in some wines. The malvidin 3-O-glucoside derivative was the major representative of each group which contained a large series of compounds derived from all major anthocyanins and reflecting their proportions in the wines. Thus, the major derived pigments detected were pyranomalvidin-3-O-glucoside (vitisin B: 0–3.6 mg·L^−1^), (epi)catechin-ethyl-malvidin-3-O-glucoside (total concentration of the four isomers = 0–2.2 mg·L^−1^), *p*-hydroxyphenylpyranomalvidin-3-*O*-glucoside (0–1.8 mg·L^−1^), catechylpyranomalvidin 3-*O*-glucoside (pinotin A: 0–0.9 mg·L^−1^), carboxypyranomalvidin-3-*O*-glucoside (vitisin A: 0–0.8 mg·L^−1^), and (epi)catechin-malvidin-3-*O*-glucoside (0–0.7 mg·L^−1^).

**Figure 2:**
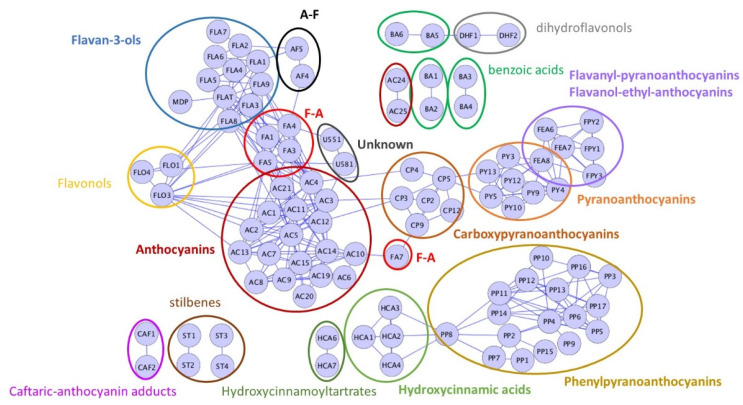
Correlation network (correlation values > 0.6) established from the polyphenol composition variables (coded as in Table S1) of the 268 wines.

The correlation network established from the phenolic composition data (Figure 2) showed close correlations within each group, and especially native anthocyanins, pyranoanthocyanins, carboxypyranoanthocyanins, phenylpyranoanthocyanins, hydroxycinnamic acids, flavonols, flavanols, methylmethine bridged anthocyanin—flavanol adducts, A-F and F-A adducts. Moreover, the concentrations of anthocyanins, flavonols, and flavanols were correlated, reflecting the extent of extraction in the wine-making process. Flavanol-anthocyanin adducts were correlated with anthocyanins and with flavanol dimers and trimers and tannins determined after phloroglucinolysis, which is consistent with their formation pathway involving cleavage of flavanol oligomers and polymers [6,7,17]. A noticeable exception is (epi)catechin-malvidin 3-*O*-acetylglucoside (FA7), correlated with malvidin 3-acetylglucoside (AC9) and its carboxypyrano derivative (CP9) meaning that formation of this pigment is limited by the availability of malvidin 3-*O*-acetylglucoside. Similarly, correlations between peonidin 3-*O*-glucoside (AC4) and carboxypyranopeonidin 3-*O*-glucoside (CP4) and between petunidin 3-glucoside (AC3) and carboxypyranopetunidin 3-*O*-glucoside (CP3) may be linked to particular grape anthocyanin profiles. Anthocyanin-flavanol adducts were correlated with flavanol monomers, which are their precursors [8]. Pyranoanthocyanins (PY) and carboxypyranoanthocyanins (CP) were only weakly correlated with native anthocyanins suggesting that their formation rates are ruled by the amount of acetaldehyde and pyruvic acid, respectively. Ethyl-linked flavanol anthocyanin adducts and flavanyl-pyranoanthocyanins were strongly correlated with pyranoanthocyanins, indicating that their concentration is driven by that of their common precursor, i.e., acetaldehyde. Phenylpyranoanthocyanins were not correlated with native anthocyanins but with hydroxycinnamic acids, revealing the limiting role of the hydroxycinnamic acids in their formation. Other strong correlations were observed between dihydroflavonols (astilbin and taxifolin), between stilbenes (*cis*- and *trans*-resveratrol, *cis*- and *trans*-piceid), between hydroxycinnamoyltartartes (caftaric and coutaric acid), indicating that all members of each group are similarly impacted by grape variety, vine growing conditions, and wine making process, and between each phenolic acid ethyl ester and its phenolic acid precursor.

### 2.2. Links between Color and Phenolic Composition

#### 2.2.1. Multivariate Analysis of the L*a*b*, Enological Analysis, and Phenolic Composition Data

**Figure 3:**
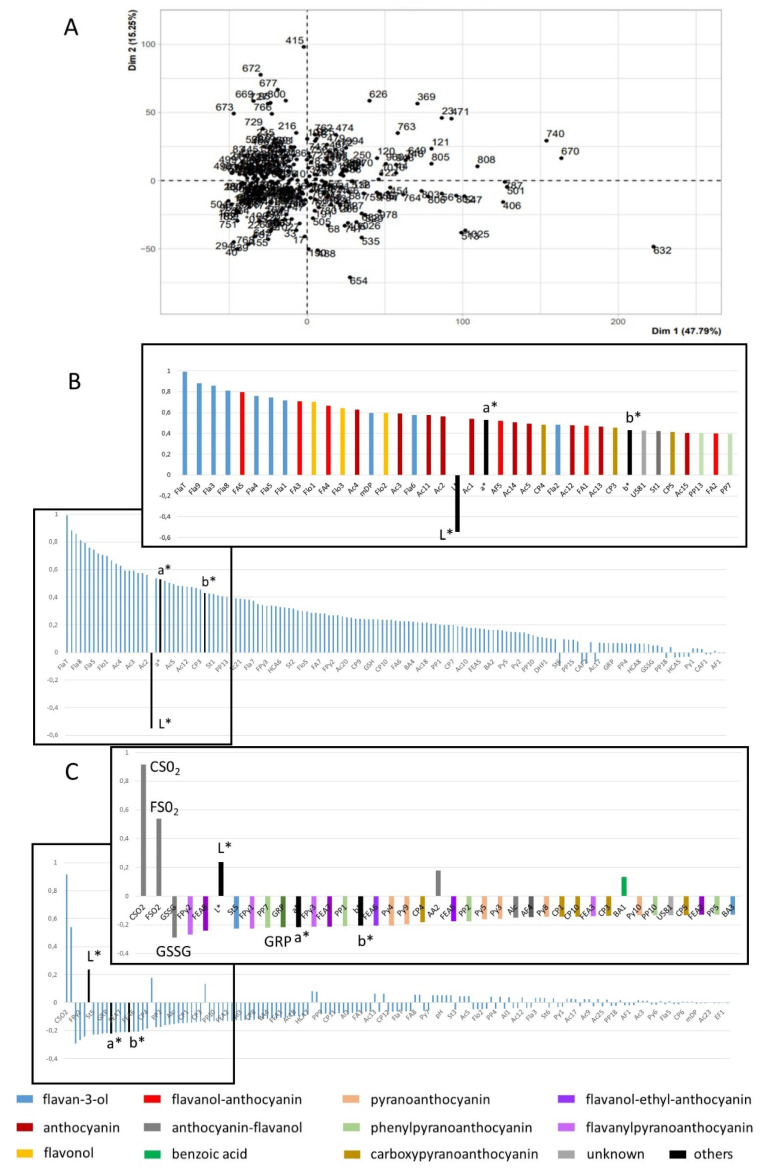
Principal component analysis of phenolic composition, enological analysis, and L*a*b* data: (**A**) Projection of the wines on the first two principal components; (**B**) loadings of variables on PC1; (**C**) loadings of variables on PC2; for (**B**,**C**), all variables in decreasing loading order and zoom presenting the first 30 variables. Variable codes provided in Table S1.

Principal component analysis (PCA) was performed on the centered MRM, oenological analysis, and L*a*b* data. Projection of the wines on the first two principal components showed large distribution along the first axis accounting for 48% of the variance (Figure 3). Loadings associated to the first PC are shown in Figure 3B. Color parameters a* and b* were associated positively and L* associated negatively with PC1, indicating that this axis was related to color. Most other variables were associated positively with PC1, with highest loadings corresponding to flavanols (monomers, dimers, trimers, total units and mDP determined by phloroglucinolysis), flavanol-anthocyanin adducts, flavonols, and anthocyanins, indicating that higher a* and b* values are related to higher flavonoid contents.

Loadings associated to the second PC, representing 15% of the variance, are shown in Figure 3C. This axis is positively associated mostly with the levels of combined and free SO_2_ and L* values and negatively with oxidized glutathione, acetaldehyde reaction products (flavanyl-pyranoanthocyanins, flavanol-ethyl-anthocyanin adducts, pyranoanthocyanins), GRP, a*, and b*.

#### 2.2.2. Composition of the Light, Intermediate, and Dark Wine Groups

In order to determine whether the links between color and composition are the same for light and dark rosé wines, three groups of wine were established based on their color intensities: light group (IC < 0.5); intermediate group (0.5 < IC < 1); dark group (IC > 1). Moreover, as all compounds of a given phenolic group share common color characteristics and the high correlations between them (Figure 2) reflect common origins (grape composition, formation process), they were considered together for further analyzing the relationships between composition and color.

Variance analysis confirmed that the three groups (Table 1) were significantly different for all measured color parameters, with CI, a*, and b* increasing, and Hue and L* decreasing from the light to dark wines. Examination of the phenolic composition of each group showed significant increases in the concentrations of anthocyanins, hydroxybenzoic acids, stilbenes, flavonols, and total flavanol units measured after phloroglucinolysis from the light to the dark group, confirming that color is primarily related to polyphenol extraction from skins. The concentrations of flavanol monomers, dimers, and trimers were significantly lower in the light group than in the other two. No significant difference was observed in the levels of hydroxycinnamic acids and hydroxycinnamoyltartrates which are more affected by the grape variety and the extent of oxidation at must stage than by maceration.

The concentrations of most derived pigments, except caftaric anthocyanin adducts, formed by enzymatic oxidation [13], also showed significant increase from the light to the dark group. A few variables showed significantly higher values in dark wines compared to the other two groups, namely ethyl-linked flavanol dimers (EF) and flavanol anthocyanin adducts (FEA), flavanylpyranoanthocyanins (FPy), and glutathione especially under its oxidized form. This might be related to higher oxidation rate, in agreement with the significantly lower free SO_2_ content of these wines.

#### 2.2.3. Links between Color and Phenolic Composition for Light, Dark and Intermediate Wines

In order to visualize the links between phenolic composition and the different color styles, PCAs were performed on the standardized MRM, oenological analysis and L*a*b* data, separately for each group.

**Figure 4:**
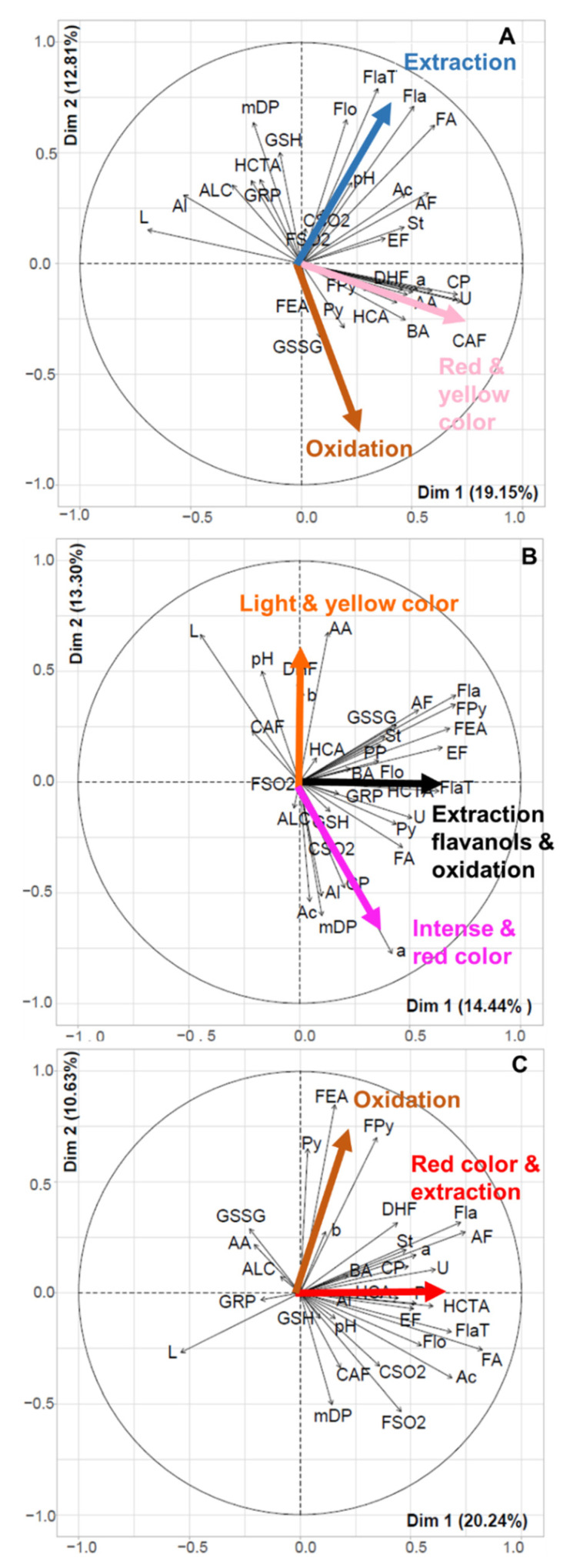
Principal component analysis of phenolic composition, enological analysis, and L*a*b* data performed on group 1 (**A**), group 2 (**B**) and group 3 (**C**); correlation circles of variables (codes provided in Table S1) on PC1 and PC2.

Projection of the variables on the first two principal components are shown for each group (Figure 4), evidencing major differences in the links of color with composition between the groups.

For group 1 (light wines), PC1 is associated positively with a* and b*, and negatively with L* and is thus a color intensity axis. Other variables positively associated to PC1 include anthocyanin derivatives, namely carboxypyranoanthocyanins and phenylpyranoanthocyanins and caftaric-anthocyanin adducts, but also unknown anthocyanin derivatives and colorless dihydroflavonols and benzoic acids. Variables reflecting the extent of extraction, such as anthocyanins, flavanols, tannin-anthocyanin and anthocyanin-tannin adducts, flavonols and stilbenes, positively contribute to both PC1 and PC2 while hydroxycinnamoyltartrates, reduced glutathione, GRP, and flavanol mDP contribute positively to PC2 and, to a lesser extent, negatively to PC1. PC2 is also negatively associated to pyranoanthocyanins, flavanol-ethyl-anthocyanin adducts and oxidized glutathione, suggesting that it is associated to oxidation.

For group 3 (dark wines), PC1 is associated negatively to L* and positively to a* while b* contributes positively to PC2. Most phenolic variables and especially anthocyanins, flavanols, hydroxycinnamic acids and all anthocyanin derivatives are also positively associated with PC1, and thus red color, except for Py, FE and FPy. These molecules, that result from reactions involving acetaldehyde, are associated positively with PC2 which is also negatively associated with sulfite concentrations and can be interpreted as an oxidation axis.

In the intermediate group, PC1, positively associated with flavanols, flavanol-anthocyanin adducts, and oxidation markers such as pyranoanthocyanins, ethyl-linked flavanols, and flavanol-ethyl-anthocyanins, reflects both extraction and oxidation. PC2, contrasting a* with L* and b*, is a red color axis, lighter wines in this group being also more orange. The red color is associated with higher concentrations of anthocyanins, carboxypyranoanthocyanins, and alcohols derived from tyrosine and tryptophan and with flavanol mDP while lighter and more yellow wines show higher values of pH, and higher concentrations of aromatic amino acids, dihydroflavonols, flavanol monomers and oligomers, anthocyanin-flavanol adducts, flavanylpyranoanthocyanins and oxidized glutathione.

## 3. Discussion

### 3.1. Rosé Wine(s) Versus Red Wine

The rosé wine collection exhibited large color differences from almost white to light red, covering the entire range of shades described in the Color Chart from the Centre du Rose^®^ (Centre Du Rosé.fr., available online: https://centredurose.fr/nuanciers-vins-roses, accessed 30 January 2022). It should be emphasized that there is no definition of rosé wine based on color and examination of color values reported in the literature for red and rosé wines show large variations and overlapping (e.g., L*: 28–70, a*: 28–52, b: 8–36 in reds [18]; L*: 38–45; a*: 48–52; b*: 24–28 in rosés [15]). However, much lighter colors have been reported in other studies (L*: 79–86, a*: 17–25, b*: 8–9 [16]; L*: 92–95, a*: 2–5, b*: 7–11 [11]) and the light “salmon” shade, which is characteristic of Provence wines, is generally the leader on the global market although consumer preferences may vary between regions [1]. The maximum color intensity value was at 2.2 and thus much lower than those reported in reds (e.g., 11–23 [19], 6–7 [20]).

**Figure 5:**
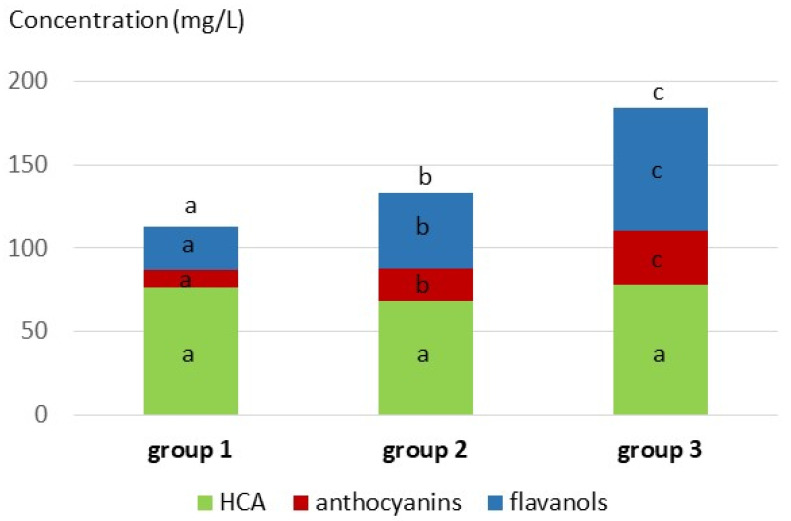
Distribution of the major families of grape phenolic compounds (i.e., anthocyanins, flavanols and hydroxycinnamic acids) in the three color groups. Different superscript letters indicate significant differences between color groups for a given parameter (ANoVA with SNK test for *p* < 0.05).

MS analysis targeting phenolic compounds also showed large composition differences. Hydroxycinnamoyltartaric acids, flavanols, and anthocyanins were the most abundant compounds detected, as shown earlier [11,14]. However, their proportions varied greatly within the collection and were significantly different in the three wine color groups (Figure 5).

Hydroxycinnamoyltartaric acids (i.e., caftaric and coutaric acids) which are abundant in the grape berry flesh and thus easily released into the must were the predominant phenolic compounds in the light rosé wines, as classically observed in white wines [21,22]. The concentrations of hydroxycinnamic acids, of GRP, and of caftaric-anthocyanin adducts showed large variations but were similar in the three color groups. Indeed, these variables depend on the grape composition in hydroxycinnamic acids and glutathione which are varietal characteristics [23] and on the extent of enzymatic oxidation during must preparation [24] but are little affected by maceration and extraction.

The concentrations of phenolic compounds localized in skins, such as anthocyanins, flavonols, flavanols and stilbenes, were lower than those generally reported in red wines and significantly increased from light to dark wines, primarily reflecting the extent of extraction from the grape berry skins although this can also be modulated by adsorption on yeast or fining treatments. For example, the low levels of coumaroylated anthocyanins (0 −11.6 mg·L^−1^) in the rosé wines are related to their low extraction rate pointed out in earlier studies [11] and recently confirmed in extraction experiments [25] but they can also result from their selective adsorption on fining agents used in rosé wine-making such as PVPP [16]. The correlation network presented in Figure 2 showed strong correlations within each group of phenolic compounds, meaning that the concentrations of all compounds of a given group are affected in the same way during the vine-growing and wine–making process. Correlations between anthocyanin, flavanol, and flavonol concentrations also indicate that extraction and possibly fining have similar impacts on these three groups. Additional correlations of derived pigments with anthocyanins and /or flavanols are related to their formation process and in a few cases, to specific anthocyanin profiles likely related to the wine variety, as explained in Section 2.1.2. In particular, formation of acetaldehyde reaction products, carboxypyranoanthocyanins and phenylpyranoanthocyanins depend on the concentrations of acetaldehyde, pyruvic acid, and hydroxycinnamates, respectively rather than on that of anthocyanins. The impact of yeast strain and fermentation conditions on the production of yeast metabolites such as acetaldehyde and pyruvic acid, sulfite consumption or production, and enzymatic activities, including cinnamate decarboxylase activity, and subsequently on phenolic and especially pigment composition is well documented [20,26]. In addition, interplay between sulfite and acetaldehyde metabolism during alcoholic fermentation has been recently demonstrated [27]. However, the impact of yeast metabolism and of chemical oxidation after fermentation on formation of acetaldehyde and pyruvic acid cannot be distinguished.

Other colorless compounds are also involved in wine discrimination along PC1 and PC2. In particular, tryptophol, hydroxytyrosol, and tyrosol are opposite to tryptophan and tyrosine, reflecting conversion of the aromatic amino acids to alcohols by yeast metabolism [28]. Aromatic alcohols are associated with lower color intensity and lower oxidation in groups 1 and 3, which may be related to the fact that fermentation forms more tyrosol and hydroxytyrosol without oxygen than with oxygen [29]. However, this was not observed in group 2, suggesting that parameters related to yeast or other microorganisms may be prevalent.

### 3.2. Pigment Composition and Color

After this general description, rosé wines were separated into three color intensity groups: light, intermediate and dark. The colors of the three wine groups are related to very different pigment composition both quantitatively and qualitatively.

The concentrations of flavanol-anthocyanin, anthocyanin-flavanol, ethyl-flavanols, anthocyanin-ethyl-flavanols, and flavanylpyranoanthocyanins adducts increased with color and were significantly higher in the dark wine group. The concentrations measured for these compounds are rather low compared to those of genuine flavanols and anthocyanins. However, the lower molecular weight (dimeric) compounds assayed in this study represent groups of related compounds formed by the same reaction and showing longer flavanol chains that are not quantified by the MRM method but contribute to the total units measured by phloroglucinolysis [17,30,31].

**Figure 6:**
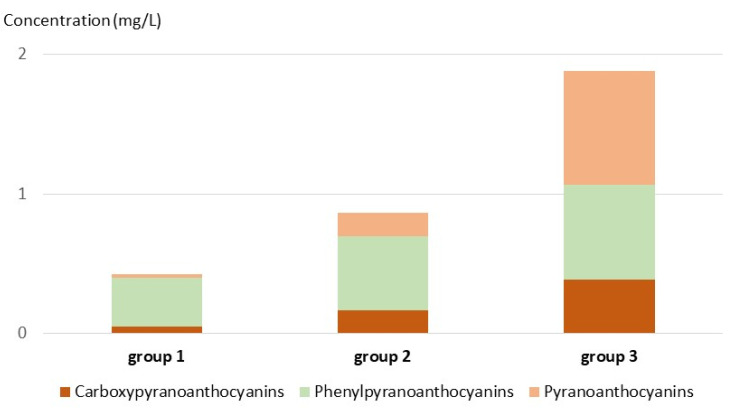
Distribution of pyranoanthocyanin, carboxypyranoanthocyanins and phenylpyranoanthocyanins in the three color groups.

The concentrations of pyranoanthocyanins, carboxypyranoanthocyanins, and phenylpyranoanthocyanins also increased significantly from the light to the dark wine group. The increase was much higher for pyranoanthocyanins and carboxypyranoanthocyanins, derived respectively from acetaldehyde and pyruvic acid, than for phenylpyranoanthocyanins, derived from hydroxycinnamates and correlated with them. Consequently, phenylpyranoanthocyanins were the major pyranoanthocyanins in wines from the light and intermediate groups whereas pyranoanthocyanins were predominant in the darker wines (Figure 6).

In light rosé wines, carboxypyranoanthocyanins and phenylpyranoanthocyanins are major contributors to the first PC, positively associated to both the red and yellow color components, along with anthocyanins and flavanol-anthocyanin dimer adducts. The first two groups of molecules show a salmon shade compared to the pink color of diluted anthocyanin solutions and have a high extinction coefficient in the visible range, compared to anthocyanins [32]. Moreover, they are resistant to sulfite bleaching [33] and hydration [10,32] reactions and thus remain under their pigmented flavylium form regardless of the pH value or the presence of sulfites whereas genuine anthocyanins and FA adducts [7] are mostly colorless in wine. This may explain their large contribution to the salmon color of the pale rosé wines, in spite of their rather low concentrations.

PCAs performed on the darker rosé wines (groups 2 and 3) show a color axis related to the red color component, contrasting L* and a*. In group 3, the red color axis (PC1) is related to anthocyanins and all derived pigments except those resulting from reactions with acetaldehyde which are associated with the yellow component on PC2, suggesting a role of oxidation in the development of browning as discussed below. Moreover, the poor representation of b* suggests that yellow hues are largely due to compounds that are not quantified by the MRM method. In group 2, the red color axis (PC2) contrasts a* with both L* and b*, meaning that the lightest wines in this group are also the most orange. Redness is associated with anthocyanins and carboxypyranoanthocyanins while lighter and more orange color is associated with higher pH values, shifting anthocyanin equilibria from red flavylium cations to colorless forms and possibly promoting oxidation. Identification of the unknown anthocyanin derivatives determined by the targeted MRM method used and of other molecules which are not analyzed by this method, is needed to characterize the yellow pigments.

### 3.3. Drivers of Rosé Wine Color

#### 3.3.1. Extraction

When considering the entire wine collection, color appears related mostly to the extent of extraction, reflected by the concentrations of flavanols and especially FlaT, anthocyanins, derived tannins and pigments, flavonols, dihydroflavonol, and stilbenes. Flavanol mDP determined by phloroglucinolysis also increases with tannin concentration, consistent with the fact that lower molecular weight flavanols are more readily extracted.

PCAs performed for each group suggest different links between extraction and color. The color of light rosé wines depends primarily on pyranoanthocyanins and is little impacted by extraction which is quite limited in these wines. In contrast, the color of dark rosé wines is determined primarily by the extent of extraction, the darkest wines of group 3 showing higher levels of all phenolic compounds and compositions closer to those of red wines.

In group 1 (light wines), mDP values contribute positively to PC2 and are thus associated with higher levels of flavanols and lower oxidation, like in the entire collection. This is not so clear in the darker wines, possibly indicating higher proportions of flavanol units arising from derived tannins in these wines. Indeed, it is well established that conversion of tannins to derived tannins through oxidation, formation of flavanol-anthocyanin adducts or condensation with acetaldehyde leads to over- or under-estimation of mDP values as some of the new bonds are resistant to phloroglucinolysis and the resulting derived units are not taken into account in the calculations [34].

#### 3.3.2. Oxidation

Oxidation appears like another important factor affecting rosé wine color. Indeed, the second axis of the PCA performed on the whole wine collection contrasts the concentration of sulfites and L* values with those of compounds resulting from reactions of anthocyanins and flavanols with acetaldehyde and oxidized glutathione, and a* and b* values and is thus associated to oxidation, enhancing color.

Higher level of pyranoanthocyanins and of other molecules arising from reactions of phenolic compounds with acetaldehyde (i.e., ethyl-flavanols, anthocyanin-ethyl-flavanols, and flavanylpyranoanthocyanins) in dark wines reflect a higher extent of oxidation in these wines, as also indicated by other variables such as the increased concentration of oxidized glutathione and lower concentration of free SO_2_. Indeed, oxidation of red [12,35] and rosé [11] wines has been reported to result first in consumption of SO_2_, followed by release of flavylium ions from anthocyanin bisulfite adducts and thus enhancement of the red color. Consumption of sulfite can result from their reaction with quinones generated by chemical oxidation of o-diphenols in the presence of metal ion catalysts [36] or with flavanols [37] and indoles [38]. Moreover, it can involve sulfite combination with carbonyl compounds such as acetaldehyde [11] and pyruvic acid [39] that are produced as fermentation byproducts but also by chemical oxidation of ethanol and malic acid, respectively [40].

Acetaldehyde is released from its sulfonates as SO_2_ is consumed and accumulates in wines submitted to oxidation once SO_2_ is depleted [35,41]. Accordingly, increased levels of acetaldehyde reaction products, including pyranoanthocyanins, flavanylpyranoanthocyanins, and ethyl-linked compounds, have been reported in wines submitted to microoxidation [31]. The last two groups of compounds also included larger oligomers which are not analyzed by the MRM method used in the present study but may contribute flavanol units after phloroglucinolysis and bias mDP calculation as explained above.

The PCAs performed on each color group confirm an impact of oxidation but suggest that different compounds and mechanisms may be involved.

In the light rosé wines, the oxidation axis (PC2) contrasts the concentrations of flavanols, flavanol-anthocyanin adducts and flavonols, flavanol mDP and, to a lesser extent, the contents of hydroxycinnamoyltartrates, glutathione, and GRP, with those of oxidized glutathione and acetaldehyde derivatives, namely pyranoanthocyanins and flavanol-ethyl-anthocyanins. Hydroxycinnamoyltartrates are known to be the major substrates of enzymatic oxidation taking place in the musts before fermentation. Their oxidation generates o-quinones which can react with glutathione to form GRP [42] or, after glutathione depletion, oxidize other o-diphenols, including GRP [43] and flavanols [44,45,46]. Enzymatic oxidation taking place before fermentation results in lower levels of all these compounds and intense browning of the must. However, the brown products formed at this stage are removed by racking and filtration steps so that they have limited impact on the final wine color [47]. Browning susceptibility of white wines has been related to their flavanol content [47,48] and several reaction mechanisms involving flavanols and resulting in browning have been described. In particular, oxidation of catechin yields yellow pigments called dehydrodicatechins A [49] and oligomers resulting from condensation with aldehydes such as glyoxylic acid [50], furfural [51] or acetaldehyde [52] proceed to intensely yellow xanthylium salts. Thus, high values of b* may be related to the presence of both orange pyranoanthocyanins and of flavanol oxidation products which are not analyzed in the targeted MRM method used but are expected to increase with both extraction and oxidation.

In the dark rosé wines, oxidation (PC2) is associated with the concentrations of FEA, Fpy, and Py and negatively with the levels of free and combined SO_2_, confirming the relevance of acetaldehyde and its reaction products as wine oxidation markers [31,53]. The yellow color component b* is also positively associated to PC2, again suggesting a role of oxidation in the development of browning.

Dihydroflavonols are not associated with extraction like other flavonoids, except in group 3, meaning that their concentration is driven by different mechanisms. In contrast, they are associated with b* in light and intermediate wines, suggesting that they may participate in oxidative browning, owing to their o-diphenolic B-ring.

## 4. Materials and Methods

### 4.1. Chemicals

Formic acid and HPLC grade methanol were purchased from VWR Prolabo (Fontenay-sous-Bois, France). Deionized water was obtained from a Milli-Q purification system (Millipore, Molsheim, France). Standard of trans-piceid was purchased from Selleckchem (Houston, TX, USA). Standards of caffeic acid, vanillic acid, gallic acid and ferulic acid were purchased from Fluka (Buchs, Switzerland). Standards of syringic acid, p-coumaric acid, protocatechuic acid, ethyl protocatechuate, tyrosol, tryptophol, tryptophan, trans-resveratrol, quercetin, catechin, epicatechin, epicatechin 3-gallate, taxifolin, oxidized and reduced glutathione were purchased from Sigma-Aldrich (St. Louis, MO, USA), as well as hydrochloric acid, phloroglucinol, L-ascorbic acid, and ammonium formiate. Standards of ethyl gallate, ethyl caffeate, tyrosine, hydroxytyrosol, epigallocatechin, malvidin 3-O-glucoside chloride, and malvidin 3,5-diglucoside chloride were purchased from Extrasynthese (Geney, France). Standards of trans-caftaric acid, quercetin glucoside and procyanidin dimers B1, B2, B3, B4, and trimer C1 were purchased from Phytolab (Vestenbergsgreuth, Germany).

### 4.2. Samples and Sampling

A set of 268 commercial rosé wines selected from the Rosé du Monde Collection of 2011 was provided by the Centre du Rosé (IFV, Vidauban, France). All of them have been sampled at the “Mondial du Rosé^®^” Contest organized in 2011 by Union de Oenologues de France, among a collection of 869 commercial rosé wines, selected so as to ensure good representation of all producing countries and regions. The wines were from 21 different countries and covered a wide range of colors. All analyses were performed in spring 2012.

Samples (250 mL) were taken immediately after opening the bottles, filtered through 0.45 µm filters (Hydrophobic Durapore, Millipore) under vacuum and degassed during 5 min under stirring. This filtered and degassed wine was then dispached in different volume aliquots for analysis. A volume of 125 mL was inerted (N_2_) and stored at 4 °C until color and enological analyses (pH, alcoholic degree, free and total sulfites content), that were performed on the same day. A 1 mL sample was filtered on a 0.2 µm regenerated cellulose membrane filter (Phenex, Phenomenex, Le Pecq, France) and directly injected in triplicate using Ultra High Performance Liquid Chromatography, coupled to triple-quadrupole Mass Spectrometry (UHPLC-QqQ-MS) in the Multiple Reaction Monitored (MRM) mode for determination of polyphenol composition. Six aliquots of 2 mL were taken to dryness under reduced pressure using Genevac (SP Scientific, Warminster, PA, USA) to perform phloroglucinolysis and stored at −80 °C until analysis.

### 4.3. Enological Analysis

Oenology parameters, namely pH, total sulfite content and free sulfite content were determined by traditional methods (Method OIV-MA-AS323-04B, OIV-MA-AS313-01). Combined sulfite content was calculated by subtracting free sulfite content from total sulfite content. Percent alcohol was determined by NIR selective absorption method using an Alcolyzer (Anton Paar, Les Ulis, France).

### 4.4. Color Analysis

The absorbance measurements were performed with an Evolution 300 UV−visible spectrophotometer (ThermoFisher, Dardilly, France) directly on the wine, using a 10 mm optical path cell. The method is described in the Compendium of International Methods of Wine and Must Analysis by the International Organisation of Vine and Wine (OIV-MA-AS2-07B). The color intensity (CI) is defined as the sum of absorbance values at 620, 520 and 420 nm corresponding respectively to blue, red and yellow wavelengths [54]. The hue value (H) is the ratio between absorbance at 420 and 520 nm.

In order to describe the color perceived by the observer, trichromatic components defined by the Commission Internationale de l’Éclairage were used (CieL*a*b*, 1976). The calculation method of the different chromatic parameters, namely, lightness L*, a* expressing the red/green component and b* expressing the yellow/blue component, is defined by OIV (OIV-MA-AS2-11).

### 4.5. Analysis of Phenolic Composition

Polyphenol composition was determined by UHPLC-MS/MS in the MRM mode, using an Acquity UHPLC system coupled to a triple quadrupole (QqQ) mass spectrometer (Waters, Saint-Quentin-en-Yvelines, France) with an electrospray ionization (ESI) source operated in both positive and negative modes. MassLynx software (Waters, Milford, MA, USA) was used to control the instruments and to acquire the data. Then, data were processed using TargetLynx software (Waters, Milford, MA, USA).

The method described by Lambert et al. [14] was applied to detect and quantify 135 phenolics, including 99 anthocyanins and derivatives (native anthocyanins, anthocyanin dimers, pyranoanthocyanins, carboxypyranoanthocyanins, phenylpyranoanthocyanins (i.e., catechyl, hydroxyphenyl, and guaiacylpyranoanthocyanins), flavanyl-pyranoanthocyanins, flavanol-anthocyanin (F−A) and anthocyanin-flavanol (A-F) dimer adducts and methylmethine-linked anthocyanin flavanol adducts (F-ethyl-A dimers); 15 phenolic acids (hydroxybenzoic acids, hydroxycinnamic acids and their methyl esters), six stilbenes, five flavonols, two dihydroflavonols, nine flavanol monomers, dimers and trimers and two F-ethyl-F dimers. Other compounds were quantified in the method such as amino acids (tyrosine and tryptophan) and the corresponding alcohols (tyrosol, hydroxytyrosol, tryptophol), reduced and oxidized glutathione and GRP (Grape reaction product, i.e., 2-S-glutathionylcaffeoyltartaric acid). Standard and sample preparations, UHPLC-MS/MS conditions, MRM parameters and calibration ranges were the same as in the method validated by Lambert et al. [14]. The variable list and codes used are presented in Table S1.

Constitutive units of flavanol derivatives released after acid-catalyzed depolymerization in the presence of phloroglucinol [55] were analysed by UHPLC-QqQ-MS in the MRM mode as described by Pinasseau et al. [56]. The phloroglucinolysis reaction was performed in triplicate on the solid obtained after evaporation of 2 mL of wine, using either 2 mL or 3 mL of a phloroglucinol (50 g/L) and ascorbic acid (10 g/L) solution in MeOH/HCl 0.2 M. The reaction was carried out for 20 min at 50 °C and stopped by placing the sample in ice and adding an identical volume of ammonium formiate buffer at 200 mM [55]. The solution obtained was centrifuged (HettichLab Technology, Tuttlingen, Germany) (15,000 rpm, 10 min, 4 °C). The supernatant was filtered through a 0.2 µm RC Membrane filter (Phenomenex, Le Pecq, France) before injection into the HPLC-QqQ-MS system. This method allows the detection and quantification of epigallocatechin, catechin, epicatechin, epicatechin-3-O-gallate (terminal units) and of their phloroglucinol adducts (extension units). Total flavanol units released by phloroglucinolysis (FlaT) were calculated and average degree of polymerisation (mDP) was evaluated as the molar ratio of the sum of all flavanol units to the sum of terminal units.

### 4.6. Chemometrics

Principal Component Analysis and ANalysis Of VAriance with a Student-Newman-Keuls post-hoc test were performed with RStudio software (www.rstudio.com, Boston, MA, USA, accessed on 30 January 2022) using Factoextra, Agricolae and Stats package. ANOVA results were considered significant at *p*-value < 0.05. The correlation network was performed using Cytoscape 3.9.0 (https://cytoscape.org/ accessed on 30 January 2022).

## 5. Conclusions

The results of the present study, performed on a large worldwide collection of commercial rosé wines, shed light on the diversity of color styles of these wines. They demonstrate large differences in phenolic and especially pigment composition between light rosé wines and wines showing more intense color, meaning that the color differences do not simply reflect dilution, and establish particular phenolic profiles related to the different color styles. Thus, the composition of light Provence style wines is reminiscent of that of white wines, with phenolic compounds represented mostly by hydroxycinnamoyltartrates while wines showing higher color intensity contain larger proportions of flavanols and anthocyanins, extracted from the grape skins.

Consequently, the color and salmon-orange hue of wines from the lighter group are primarily due to phenylpyranoanthocyanins derived from hydroxycinnamates and carboxypyranoanthocyanins formed from pyruvic acid, a yeast metabolite. Indeed, these compounds are orange pigments and, unlike their anthocyanin precursors, hundred percent colored in mildly acidic and dilute media such as light rosé wine. The more intense and redder color of wines from the intermediate group is associated to higher levels of anthocyanins and carboxypyranoanthocyanins and lower pH values, while that of the darkest wines is impacted by pigments derived from anthocyanins and flavanols, including derived phenolic compounds that are not among the targets of the MRM method used in the present study. Interpretation of the yellow component of color, b*, is more complex. For the intermediate group, it is associated with low color intensity, high pH values, and high levels of compounds such as dihydroflavonols, aromatic amino acids, flavanols and anthocyanin-flavanol adducts, which are not themselves colored but, in particular under oxidative conditions, may be the precursors of yellow pigments that require further investigation. For the darker wines, yellowness appears associated to oxidation, and especially products derived from reactions of acetaldehyde with anthocyanins and flavanols which can be considered as markers of larger families of related compounds. Untargeted metabolomics approaches may help identify these pigments.

Beyond their implications for color and thus quality of rosés wines, the phenolic composition profiles obtained in the present study may be of use for authentication of the varietal or geographical origins of these wines.

## Figures and Tables

**Table 1:** Comparison of the phenolic composition, enological analysis, spectrophotometry and color data of the 3 color groups.

Variable	Light	Intermediate	Dark
Phenolic Compounds (mg·L^−1^)	Mean ± SD (*n* = 114)	Mean ± SD (*n* = 101)	Mean ± SD (*n* = 53)
Benzoic acids and ethyl esters (BA 1–6)	6.2 ± 6.0 ^a^	9.9 ± 7.1 ^b^	13.2 ± 7.5 ^c^
Hydroxycinnamic acids and ethyl esters (HCA 1–5)	3.6 ± 2.8 ^a^	4.2 ± 5.5 ^a^	3.9 ± 3.6 ^a^
Hydroxycinnamoyl tartaric acids (HCA 6–8)	53.4 ± 27.2 ^a^	47.3 ± 21.0 ^a^	50.0 ± 26.3 ^a^
2-S-glutathionylcaffeoyltartaric acid (GRP)	19.8 ± 12.5 ^a^	16.8 ± 10.7 ^a^	24.6 ± 18.6 ^b^
Stilbenoids (St 1–6)	3.2 ± 2.4 ^a^	4.6 ± 3.4 ^b^	5.9 ± 6.4 ^c^
Flavonols (Flo 1–5)	0.7 ± 1.5 ^a^	1.4 ± 1.7 ^b^	2.1 ± 1.6 ^c^
Dihydroflavonols (DHF 1–2)	0.9 ± 0.8 ^a^	1.6 ± 1.8 ^b^	0.9 ± 0.7 ^a^
Flavanols monomers and oligomers (Fla 1–9)	19.2 ± 19.6 ^a^	32.3 ± 26.7 ^b^	33.6 ± 22.7 ^b^
Total flavanol units after phloroglucinolysis (FlaT)	25.3 ± 27.6 ^a^	45.3 ± 31.8 ^b^	73.5 ± 45.5 ^c^
Mean degree of polymerisation (mDP)	2.4 ± 0.6 ^a^	2.7 ± 0.9 ^a^	3.5 ± 1.3 ^b^
Anthocyanins (Ac 1–25)	10.6 ± 6.2 ^a^	19.2 ± 12.7 ^b^	32.2 ± 19.1 ^c^
Pyranoanthocyanins (Py 1–13)	0.02 ± 0.05 ^a^	0.17 ± 0.30 ^b^	0.82 ± 0.95 ^c^
Carboxypyranoanthocyanins (CP 1–12)	0.05 ± 0.04 ^a^	0.17 ± 0.15 ^b^	0.39 ± 0.25 ^c^
Flavanol-anthocyanin adducts (FA 1–8)	0.05 ± 0.07 ^a^	0.13 ± 0.11 ^b^	0.30 ± 0.26 ^c^
Anthocyanin-flavanol adducts (A-type) (AF 1–5)	0.001 ± 0.005 ^a^	0.005 ± 0.011 ^b^	0.010 ± 0.015 ^c^
Caftaric-anthocyanin adducts (CAF 1–2)	0.02 ± 0.04 ^a^	0.03 ±0.04 ^a^	0.02 ±0.03 ^a^
Ethyl-flavanol dimers (EF 1–2)	0.001 ± 0.003 ^a^	0.006 ± 0.024 ^ab^	0.008 ± 0.011 ^b^
Flavanol-ethyl-anthocyanins (FEA 1–8)	0.003 ± 0.023 ^a^	0.064 ± 0.319 ^a^	0.205 ± 0.351 ^b^
Phenylpyranoanthocyanins (PP 1–18)	0.35 ± 0.41 ^a^	0.53 ± 0.48 ^b^	0.68 ± 0.59 ^c^
Flavanyl-pyranoanthocyanins (FPy 1–3)	0.002 ± 0.007 ^a^	0.010 ± 0.037 ^ab^	0.016 ± 0.044 ^b^
Unknown 551 & 581 (U 551 & 581)	0.016 ± 0.020 ^a^	0.038 ± 0.037 ^b^	0.092 ± 0.066 ^c^
Alcohols (Al 1–3)	13.6 ± 6.3 ^a^	11.5 ± 6.1 ^a^	15.8 ± 9.3 ^b^
Amino acids (AA 1–2)	7.9 ± 3.5 ^a^	8.6 ± 4.1 ^a^	6.4 ± 3.3 ^b^
Glutathione (GSH)	0.06 ± 0.05 ^a^	0.06 ± 0.07 ^a^	0.09 ± 0.09 ^b^
Oxidized glutathione (GSSG)	0.06 ± 0.13 ^a^	0.18 ± 0.55 ^a^	0.91 ± 3.21 ^b^
**Enological analysis**			
pH	3.4 ± 0.1 ^a^	3.4 ± 0.2 ^a^	3.4 ± 0.2 ^a^
Free sulfites content (FSO_2_)	25 ± 20 ^a^	21 ± 13 ^a^	16 ± 9 ^b^
Combined sulfites content (CSO_2_)	75 ± 21 ^a^	78 ± 23 ^a^	74 ± 23 ^a^
% alcohol (ALC)	12.7 ± 0.9 ^a^	12.7 ± 1.2 ^a^	13.1 ± 0.8 ^b^
**Color analysis**			
L*	93.2 ± 2.2 ^a^	84.6 ± 3.5 ^b^	70.6 ± 5.5 ^c^
a*	7.0 ± 3.2 ^a^	17.3 ± 5.71 ^b^	34.7 ± 7.6 ^c^
b*	10.30 ± 2.68 ^a^	15.62 ± 3.32 ^b^	20.0 ± 4.2 ^c^
Color intensity (CI)	0.33 ± 0.10 ^a^	0.69± 0.14 ^b^	1.36 ± 0.31 ^c^
Hue (H)	1.50 ± 0.47 ^a^	1.16 ± 0.26 ^b^	0.88 ± 0.16 ^c^

Different superscript letters indicate significant differences between color groups for a given parameter (ANoVA with SNK test for *p* < 0.05).

## Data Availability

The data presented in this study are available in the Supplementary Information Section.

## Supplementary Materials

The following supporting information can be downloaded online. Table S1: List of variables and codes.docx, Table S2: RoseDataset.xls.

## Sample Availability

Samples of the compounds are not available from the authors.
