# Supplementary material for: Elucidating the Color of Rosé Wines Using Polyphenol-Targeted Metabolomics
Source: Molecules. 2022 Feb 17;27(4):1359. doi: 10.3390/molecules27041359 (PMC8874620; doi:10.3390/molecules27041359)
Supplement: Supplementary file 1 [file molecules-27-01359-s001.zip › TableS1-List of variables and codes.pdf]

Phenolic compounds	codes
**Benzoic acids and ethyl esters (mg/L)**	
protocatechuic acid	BA1
protocatechuic acid ethyl ester	BA2
gallic acid	BA3
gallic acid ethyl ester	BA4
vanillic acid	BA5
syringic acid	BA6
**Hydroxycinnamic acids (free and ethyl esters) (mg/L)**	
p-coumaric acid	HCA1
p-coumaric acid ethyl ester (mg/L caffeic acid ethyl ester equivalent)	HCA2
caffeic acid	HCA3
caffeic acid ethyl ester	HCA4
ferulic acid	HCA5
**Hydroxycinnamoyl tartrates (mg/L)**	
p-coumaroyltartaric acid (mg/L caffeoyltartaric acid equivalent)	HCA6
caffeoyltartaric acid	HCA7
feruloyltartaric acid (mg/L caffeoyltartaric acid equivalent)	HCA8
2-S-glutathionylcaffeoyltartaric acid (Grape Reaction Product) (mg/L caffeoyltartaric acid equivalent)	GRP
**Stilbenoids (mg/L)**	
trans-piceid	ST1
cis-piceid (mg/L trans-piceid equivalent)	ST2
trans-resveratrol	ST3
cis-resveratrol (mg/L trans-resveratrol equivalent)	ST4
resveratrol dimer 1 (mg/L trans-resveratrol equivalent)	ST5
resveratrol dimer 2 (mg/L trans-resveratrol equivalent)	ST6
**Flavonols (mg/L)**	
quercetin 3-O-glucuronide (mg/L quercetin 3-O-glucoside equivalent)	FLO1
quercetin 3-O-glucoside	FLO2
myricetin 3-O-glucuronide (mg/L quercetin 3-O-glucoside equivalent)	FLO3
myricetin 3-O-glucoside (mg/L quercetin 3-O-glucoside equivalent)	FLO4
quercetin	FLO5
**Dihydroflavonols (mg/L)**	
taxifolin	DHF1
astilbin (mg/L quercetin 3-O-glucoside equivalent)	DHF2
**Flavan-3-ol monomers and oligomers (mg/L)**	
catechin	FLA1

epicatechin	FLA2
procyanidin dimer B1 (mg/L procyanidin dimer B2 equivalent)	FLA3
procyanidin dimer B2	FLA4
procyanidin dimer B3 (mg/L procyanidin dimer B2 equivalent)	FLA5
procyanidin dimer B4 (mg/L procyanidin dimer B2 equivalent)	FLA6
procyanidin dimer gallate (mg/L catechin equivalent)	FLA7
procyanidin trimer 1 (mg/L catechin equivalent)	FLA8
procyanidin trimer 2 (mg/L catechin equivalent)	FLA9

Flavan-3-ol phloroglucinolysis data

total flavanol units (phloroglucinolysis) (mg/L)	FLAT
mean degree of polymerisation (phloroglucinolysis)	mDP

Anthocyanins (mg/L malvidin 3-O-glucoside equivalent or mg/L malvidin 3,5-diglucoside equivalent *)

cyanidin 3-O-glucoside	AC1
delphinidin 3-O-glucoside	AC2
petunidin 3-O-glucoside	AC3
peonidin 3-O-glucoside	AC4
malvidin 3-O-glucoside	AC5
cyanidin 3-O-acetylglucoside	AC6
delphinidin 3-O-acetylglucoside	AC7
petunidin 3-O-acetylglucoside	AC8
peonidin 3-O-acetylglucoside	AC9
malvidin 3-O-acetylglucoside	AC10
cyanidin 3-O-coumaroylglucoside	AC11
delphinidin 3-O-coumaroylglucoside	AC12
petunidin 3-O-coumaroylglucoside	AC13
peonidin 3-O-coumaroylglucoside	AC14
malvidin 3-O-coumaroylglucoside	AC15
cyanidin 3-O-caffeoylglucoside	AC16
delphinidin 3-O-caffeoylglucoside	AC17
petunidin 3-O-caffeoylglucoside	AC18
peonidin 3-O-caffeoylglucoside	AC19
malvidin 3-O-caffeoylglucoside	AC20
cyanidin 3,5-diglucoside*	AC21
delphinidin 3,5-diglucoside*	AC22
petunidin 3,5-diglucoside*	AC23
peonidin 3,5-diglucoside*	AC24
malvidin 3,5-diglucoside*	AC25

Pyranoanthocyanins (mg/L malvidin-3-O-glucoside equivalent)

pyranocyanidin 3-O-glucoside	PY1
pyranodelphinidin 3-O-glucoside	PY2
pyranopetunidin 3-O-glucoside	PY3
pyranopeonidin 3-O-glucoside	PY4
Pyranomalvidin 3-O-glucoside = vitisin B	PY5

pyranocyanidin 3-O-acetylglucoside	PY6
pyranodelphinidin 3-O-acetylglucoside	PY7
pyranopetunidin 3-O-acetylglucoside	PY8
pyranopeonidin 3-O-acetylglucoside	PY9
pyranomalvidin 3-O-acetylglucoside	PY10
pyranopetunidin 3-O-coumaroylglucoside	PY11
pyranopeonidin 3-O-coumaroyl-glucoside	PY12
pyranomalvidin 3-O-coumaroylglucoside	PY13
**Carboxypyranoanthocyanins (mg/L malvidin-3-O-glucoside equivalent)**	
carboxypyranocyanidin 3-O-glucoside	CP1
carboxypyranodelphinidin 3-O-glucoside	CP2
carboxypyranopetunidin 3-O-glucoside	CP3
carboxypyranopeonidin 3-O-glucoside	CP4
carboxypyranomalvidin 3-O-glucoside = vitisin A	CP5
carboxypyranodelphinidin 3-O-acetylglucoside	CP6
carboxypyranopetunidin 3-O-acetylglucoside	CP7
carboxypyranopeonidin 3-O-acetylglucoside	CP8
carboxypyranomalvidin 3-O-acetylglucoside	CP9
carboxypyranopetunidin 3-O-coumaroylglucoside	CP10
carboxypyranopeonidin 3-O-coumaroylglucoside	CP11
carboxypyranomalvidin 3-O-coumaroylglucoside	CP12
**Flavanol-anthocyanin adducts (mg/L malvidin-3-O-glucoside equivalent)**	
(epi)catechin-cyanidin 3-O-glucoside	FA1
(epi)catechin-delphinidin 3-O-glucoside	FA2
(epi)catechin-petunidin 3-O-glucoside	FA3
(epi)catechin-peonidin 3-O-glucoside	FA4
(epi)catechin-malvidin 3-O-glucoside	FA5
(epi)catechin-peonidin 3-O-acetylglucoside	FA6
(epi)catechin-malvidin 3-O-acetylglucoside	FA7
(epi)catechin-malvidin 3-O-coumaroylglucoside	FA8
**Anthocyanin-flavanol adducts (A-type) (mg/L malvidin-3-O-glucoside equivalent)**	
cyanidin 3-O-glucoside -(epi)catechin (A-type)	AF1
delphinidin 3-O-glucoside -(epi)catechin (A-type)	AF2
petunidin 3-O-glucoside -(epi)catechin (A-type)	AF3
peonidin 3-O-glucoside -(epi)catechin (A-type)	AF4
malvidin 3-O-glucoside-(epi)catechin (A-type)	AF5
**Caftaric-anthocyanin adducts (mg/L malvidin-3-O-glucoside equivalent)**	
caftaric-peonidin 3-O-glucoside	CAF1
caftaric-malvidin 3-O-glucoside	CAF2
**Ethyl-flavanol dimers (mg/L catechin equivalent)**	
(epi)catechin-ethyl-(epi)catechin-1	EF1

(epi)catechin-ethyl-(epi)catechin-2	EF2
**Flavanol-ethyl-anthocyanins (mg/L malvidin-3-O-glucoside equivalent)**	
(epi)catechin-ethyl-peonidin 3-O-glucoside-1	FEA1
(epi)catechin-ethyl-peonidin 3-O-glucoside-2	FEA2
(epi)catechin-ethyl-peonidin 3-O-glucoside-3	FEA3
(epi)catechin-ethyl-peonidin 3-O-glucoside-4	FEA4
(epi)catechin-ethyl-malvidin 3-O-glucoside-1	FEA5
(epi)catechin-ethyl-malvidin 3-O-glucoside-2	FEA6
(epi)catechin-ethyl-malvidin 3-O-glucoside-3-4	FEA7
(epi)catechin-ethyl-malvidin 3-O-coumaroylglucoside	FEA8
**Phenylpyranoanthocyanins (mg/L malvidin-3-O-glucoside equivalent)**	
p-hydroxyphenylpyranopeonidin 3-O-glucoside	PP1
p-hydroxyphenylpyranomalvidin 3-O-glucoside	PP2
p-hydroxyphenylpyranopeonidin 3-O-acetylglucoside	PP3
p-hydroxyphenylpyranomalvidin 3-O-acetylglucoside	PP4
p-hydroxyphenylpyranopeonidin 3-O-coumaroylglucoside	PP5
p-hydroxyphenylpyranomalvidin 3-O-coumaroylglucoside	PP6
p-hydroxyphenylpyranopetunidin 3-O-glucoside + catechylpyranopeonidin 3-O-glucoside	PP7
catechylpyranomalvidin 3-O-glucoside (pinotin A)	PP8
catechylpyranopetunidin 3-O-acetylglucoside	PP9
catechylpyranopeonidin 3-O-acetylglucoside+p-hydroxyphenylpyranopetunidin 3-O-acetylglucoside	PP10
catechylpyranomalvidin 3-O-acetylglucoside	PP11
catechylpyranopetunidin 3-O-coumaroylglucoside	PP12
catechylpyranopeonidin 3-O-coumaroylglucoside+p-hydroxyphenylpyranopetunidin 3-O-coumaroylglucoside	PP13
catechylpyranomalvidin 3-O-coumaroylglucoside	PP14
guaiacylpyranomalvidin 3-O-glucoside	PP15
guaiacylpyranomalvidin 3-O-acetylglucoside	PP16
guaiacylpyranomalvidin 3-O-coumaroylglucoside	PP17
syringylpyranomalvidin 3-O-glucoside	PP18
**Flavanyl-pyranoanthocyanins (mg/L malvidin-3-O-glucoside equivalent)**	
pyranopeonidin 3-O-glucoside-(epi)catechin	FPY1
pyranomalvidin 3-O-glucoside-(epi)catechin	FPY2
pyranomalvidin 3-O-coumaroylglucoside-(epi)catechin	FPY3
**Others**	
unknown 581 (mg/L malvidin-3-O-glucoside equivalent)	U581
unknown 551 (mg/L malvidin-3-O-glucoside equivalent)	U551
tryptophol	Al1
tyrosol	Al2
hydroxytyrosol	Al3
tyrosine	AA1
tryptophane	AA2
glutathione (GSH)	GSH

oxidized glutathione (GSSG)	GSSG
**Enological variables**	
pH	pH
Free SO2	FSO2
Combined SO2	CSO2
Alcohol (%)	ALC
**Color variables**	
Color intensity	CI
Hue	H
L*	L
a*	a
b*	b
